# Global Paths to AFU Designation: Examples From the Asian and North American Regions

**DOI:** 10.1093/geroni/igaf122.1001

**Published:** 2025-12-31

**Authors:** Katarina Friberg-Felsted, Jacqueline Eaton, M Aaron Guest

## Abstract

The Age-Friendly University Global Secretariat promotes and processes applications to the Age-Friendly University Global Network (AFUGN). Membership indicates institutional commitment to endorsing the 10 Principles and implementing age-inclusive practices to promote older adult value and engagement in academic settings. New this year is the Guidebook For Endorsing the 10 Principles of an Age-Friendly University, created to assist institutions of higher education in becoming a designated Age-Friendly University. This symposium provides an overview of the endorsement guidebook, addressing the four steps of the process, and examples from a variety of institutions who will share their individual journeys in obtaining the AFU designation. Anderson, representing the University of Mississippi, will describe the challenges and opportunities in seeking AFU membership at an R1 university located in a rural, diversely populated state. Borrero will demonstrate the alignment between the AFU application and the University of Indianapolis’s new mission and vision “Anytime. Anywhere. For Life.” The holistic approach taken by the Chinese University of Hong Kong, including the Actionable Social Knowledge initiative, will be outlined by Chen. Perry will explore the team-based, goal-oriented, principle driven approach taken at Frontier Nursing University which led to sustainable system level efforts. Aaron Guest, Chair of the Age Friendly University Global Secretariat, will serve as discussant.

